# MsrA Overexpression Targeted to the Mitochondria, but Not Cytosol, Preserves Insulin Sensitivity in Diet-Induced Obese Mice

**DOI:** 10.1371/journal.pone.0139844

**Published:** 2015-10-08

**Authors:** JennaLynn Hunnicut, Yuhong Liu, Arlan Richardson, Adam B. Salmon

**Affiliations:** 1 The Sam and Ann Barshop Institute for Longevity and Aging Studies, The University of Texas Health Science Center at San Antonio, San Antonio, Texas, United States of America; 2 Department of Molecular Medicine, The University of Texas Health Science Center at San Antonio, San Antonio, Texas, United States of America; 3 Geriatric Research, Education and Clinical Center, South Texas Veterans Health Care System, San Antonio, Texas, United States of America; 4 Reynolds Oklahoma Center on Aging, University of Oklahoma Health Sciences Center and Oklahoma City VA Medical Center, Oklahoma, Oklahoma, United States of America; Pennington Biomedical Research Center, UNITED STATES

## Abstract

There is growing evidence that oxidative stress plays an integral role in the processes by which obesity causes type 2 diabetes. We previously identified that mice lacking the protein oxidation repair enzyme methionine sulfoxide reductase A (MsrA) are particularly prone to obesity-induced insulin resistance suggesting an unrecognized role for this protein in metabolic regulation. The goals of this study were to test whether increasing the expression of MsrA in mice can protect against obesity-induced metabolic dysfunction and to elucidate the potential underlying mechanisms. Mice with increased levels of MsrA in the mitochondria (TgMito MsrA) or in the cytosol (TgCyto MsrA) were fed a high fat/high sugar diet and parameters of glucose homeostasis were monitored. Mitochondrial content, markers of mitochondrial proteostasis and mitochondrial energy utilization were assessed. TgMito MsrA, but not TgCyto MsrA, mice remain insulin sensitive after high fat feeding, though these mice are not protected from obesity. This metabolically healthy obese phenotype of TgMito MsrA mice is not associated with changes in mitochondrial number or biogenesis or with a reduction of proteostatic stress in the mitochondria. However, our data suggest that increased mitochondrial MsrA can alter metabolic homeostasis under diet-induced obesity by activating AMPK signaling, thereby defining a potential mechanism by which this genetic alteration can prevent insulin resistance without affecting obesity. Our data suggest that identification of targets that maintain and regulate the integrity of the mitochondrial proteome, particular against oxidative damage, may play essential roles in the protection against metabolic disease.

## Introduction

Environmental factors, such as obesity, and genetic factors are both causative for the underlying pathophysiology of T2DM, particularly in the development of hyperglycemia and insulin resistance. However, there remains only a vague understanding of the basic mechanisms through which these factors promote such metabolic dysfunctions. There is growing evidence from clinical and basic research suggesting that oxidative stress plays a defining role in this process and it is now well-recognized that there is an association between elevated levels of oxidative stress and damage *in vivo* and the clinical assessment of T2DM [1–3]. Moreover, markers of oxidative stress have been reported to increase even in those with pre-diabetes, suggesting that an elevation of oxidative stress precedes the clinical diagnosis of diabetes [3]. In mice, genetic or pharmaceutical reduction of oxidative stress has been shown to be sufficient to reduce the severity of, or even prevent, hyperglycemia and insulin resistance caused by high fat feeding [4–7]. While these results point to a likely role for oxidative stress in the pre-clinical stages of T2DM, the use of broad-range antioxidants has limited clinical benefits in treating T2DM or many of its complications [8]. One possible interpretation of this lack of effect may be that the cellular oxidative damage caused by oxidative stress prior to the diagnosis of T2DM may drive the diminished metabolic response associated with obesity. Because antioxidants simply alleviate oxidative stress, perhaps identifying ways to repair or remove this damage may then have therapeutic benefits for metabolic disease that haven’t been realized using current approaches.

The protein oxidation repair enzyme methionine sulfoxide reductase A (MsrA) has been linked with the development of metabolic dysfunction both correlatively and causatively. MsrA is one of two mammalian methionine sulfoxide reductase isoforms that play a critical role in maintaining the proteome in a relatively reduced state by catalytically repairing oxidized residues of the essential amino acid methionine [9]. The sulfur-containing side chain of methionine is extremely sensitive to oxidation which can directly and detrimentally alter the conformation of many proteins due to the change in hydrophobicity that occurs during methionine oxidation [10]. Interestingly, multiple genome-wide association studies (GWAS) have identified single nucleotide polymorphisms within a genetic loci containing the human MSRA gene that are associated with central adiposity and BMI, changes in fat distribution, and metabolic syndrome in studies of multiple different populations [11–14]. In addition, both genetic and diet-induced obesity in rats promotes a significant reduction of MsrA activity in visceral adipose tissue [15]. However, until recently, it remained unclear whether these association were driven by a direct anti-diabetogenic role for MsrA. We recently showed that the lack of MsrA in mice (MsrA KO) directly impairs the regulation of glucose metabolism in that MsrA KO mice are strikingly more susceptible to high fat diet-induced insulin resistance, at least in part by regulating accumulation of oxidative damage [16].

Given these findings, we hypothesized that an increase in MsrA may be beneficial to glucose metabolism and protect against the metabolic dysfunctions caused by obesity. Normally, approximately 75% of total cellular mammalian MsrA is targeted to the cytosol and the remaining 25% to the mitochondria with both pools originating from a single MsrA gene that undergoes alternative translation [17, 18]. To dissect the role of the isoforms of MsrA in this study, we utilized two transgenic mouse models generated to overexpress levels of MsrA in different sub-cellular compartments. One mouse model targets this increased MsrA expression preferentially to the mitochondria (TgMito MsrA) and the second targets MsrA preferentially to the cytosol (TgCyto MsrA) [19, 20]. In this study, we identified that mitochondrial MsrA protects against obesity-induced metabolic dysfunction, further highlighting the importance of mitochondria in the regulation of glucose metabolism under metabolic stress and identifying a novel role for MsrA in this process.

## Methods

### Animals

All procedures involving mice were approved by the Subcommittee for Animal Studies at the Audie L. Murphy Veterans Administration Hospital at San Antonio and the Institutional Animal Care and Use Committee at the University of Texas Health Science Center at San Antonio. TgMito MsrA mice used in this study were previously identified as TgMito_MsrAMTS and mitochondrial-targeting of overexpressed transgene was accomplished utilizing the preferential translation from the native mitochondrial targeting sequence [20]. TgCyto MsrA mice in this study were previously identified as TgCyto_Myr and cytosolic-targeting of overexpressed transgene was accomplished by deletion of the mitochondrial targeting sequence [20]. The maintenance and characterization of MsrA knockout mice (MsrA KO) have been previously described [16]. TgMito X MsrA KO mice were generated by targeted breeding of MsrA KO and TgMito MsrA mice. All strains of mice have been maintained in the C57BL/6J genetic background. Mice were genotyped for presence of transgene (or neo cassette in the case of MsrA KO) and overexpression/knockout was confirmed by immunoblot. Mice were maintained at a density of 3–4 mice/cage. For feeding and insulin resistance studies, male mice were used after reaching adulthood (between 6–8 months of age).

### High fat/high sugar diets

All high fat/high sugar (hereafter abbreviated HFD) studies utilized male mice and diet studies were initiated at approximately 6–8 months of age. Prior to dietary studies, mice were maintained on a standard NIH-31 chow diet (Harlan Teklad, Madison, WI, USA) provided ad libitum. Control-fed mice in this study were maintained on this standard chow throughout. For HFD studies, male mice at 6–8 months were fed a commercially available, defined diet containing 45% kcal from fat the source of which was primarily lard (58V8, TestDiet, St. Louis MO). Both chow and HFD were provided ad libitum and food consumption and body weight were monitored biweekly. Mice were fed HFD for a total period of 13 weeks; at 12 weeks a glucose tolerance test was performed and at 13 weeks an insulin tolerance test was performed (methods described below). Mice were sacrificed during week 13 of HFD feeding.

### Insulin tolerance tests and blood chemistry

For insulin tolerance tests, mice were fasted for 6 hours during the light cycle (09:00–15:00) prior to intraperitoneal (ip) injection of 1U of insulin (Novalin R; Novo Nordisk, Princeton, NJ, USA) per kilogram of body weight. Blood glucose was measured at indicated time points after injection by ONE Touch Ultra handheld glucometer. For measurements of metabolites, blood plasma was collected at sacrifice of mice fasted overnight (18:00–09:00) and measurements of insulin (Crystal Chem, Downer’s Grove, IL), adiponectin (EMD Millipore, Billerica, MA), and leptin (EMD Millipore) were performed by ELISA following manufacturer’s instructions. HOMA-IR were calculated using HOMA2 calculator available from University of Oxford.

### Subcellular fractionization and immunoblots

Subcellular fractionations were performed by enrichment centrifugation of samples isolated from freshly collected whole hind-limb skeletal muscle. We used a modified method of mitochondrial isolation previously described [21]. After excision, tissues were minced and homogenized in Chappell-Perry buffer I [100 mM KCl, 50 mM 3-propanesulfonic acid (MOPS), 1mM ethylenediaminetetraacetic acid (EDTA), 5mM MgSO_4_, 1mM ATP, pH 7.4] with the protease nagarse then centrifuged 10 minutes at 1,000 x g. A portion of the supernatant (1,000s) was retained for assessment of total cellular MsrA content and the remainder passed through cheesecloth and centrifuged at 10,000 x g for 10 minutes. The final pellet containing the mitochondrial fraction (10,000p) and the supernatant containing cytosolic components (10,000s) were collected; the (10,000p) fraction was solubilized in Chappell-Perry buffer I containing 1% Tween-20 and protease inhibitors in preparation for immunoblot. For all other immunoblots, total protein extracts were isolated from liver, skeletal muscle (gastrocnemius) and visceral fat (epigonadal depot) tissue that had been snap frozen in liquid nitrogen after mouse sacrifice and stored at -80°C until use. Prior to sacrifice, mice had either been (1) fasted overnight and given an ip injection of insulin (1U/kg body wt) or (2) fasted overnight and given an ip injection of saline. Mice were then sacrificed 15 minutes after injection. Protein extracts were homogenized in RIPA buffer with additional protease and phosphatase inhibitors (Thermo Scientific, Rockford, IL, USA), centrifuged at 14,000g at 4°C for 15 minutes, and then stored at -80°C until needed. Equal amounts of protein samples were separated electrophoretically by SDS-PAGE and then transferred to polyvinylidene difluoride membrane (Millipore, Billerica, MA, USA). Primary antibodies and their sources used in this study: MsrA, GAPDH, mitochondrial complex I subunit 20 (CI-20), LonP, HtrA2, Hsp60, Grp75, Mitosciences OxPhos Cocktail (Abcam, Cambridge MA) and phosphor-Akt (Ser473), phospho-GSK-3α Ser21), Akt, GSK-3α, phospho-ACC (Ser79), ACC, phospho-AMPKβ1 (Ser108) and AMPKβ1/2 (Cell Signaling, Beverly MA). All alkaline phosphatase-conjugated secondary antibodies (anti-rabbit and anti-mouse) were purchased through Santa Cruz Biotechnologies (Santa Cruz, CA, USA). Protein bands on immunoblots were detected using ECL reagent (Thermo Scientific) and analyzed using ImageJ.

### Citrate synthase activity

Citrate synthase catalyzes the reaction of Acetyl CoA and oxaloacetate to form citrate and CoA-SH. Tissue homogenates prepared in RIPA buffer were assayed in Triethanolamine-HCl-buffer (pH 8.0) containing Acetyl-CoA, oxaloacetic acid, and DTNB [5,5'-dithiobis-(2-nitrobenzoic acid] (Sigma-Aldrich, St. Louis MO) to assay citrate synthase activity in a 96 well plate. To account for background reactions, an initial reaction performed in the absence of oxaloacetic acid was performed and subtracted from the final values of the reactions containing oxaloacetic acid. The CoA-SH reacts with DTNB to produce the yellow color which can be measured at 412 nm with kinetic reading on Spectra Max plate reader with the rate of color development being proportional to citrate synthase activity. The data was normalized by the amount of protein used during the assay and presented as nanomole per milligram protein per minute.

### Statistical analyses

Data were analyzed by one way or two way ANOVA with post-hoc analysis by Holm-Sidak or by Student’s t-test where appropriate. Data reaching p < 0.05 (with adjustment for multiple comparison) were considered statistically significant.

## Results

### Mitochondrial, but not cytosolic, MsrA overexpression prevents obesity-induced insulin resistance

**Fig 1:**
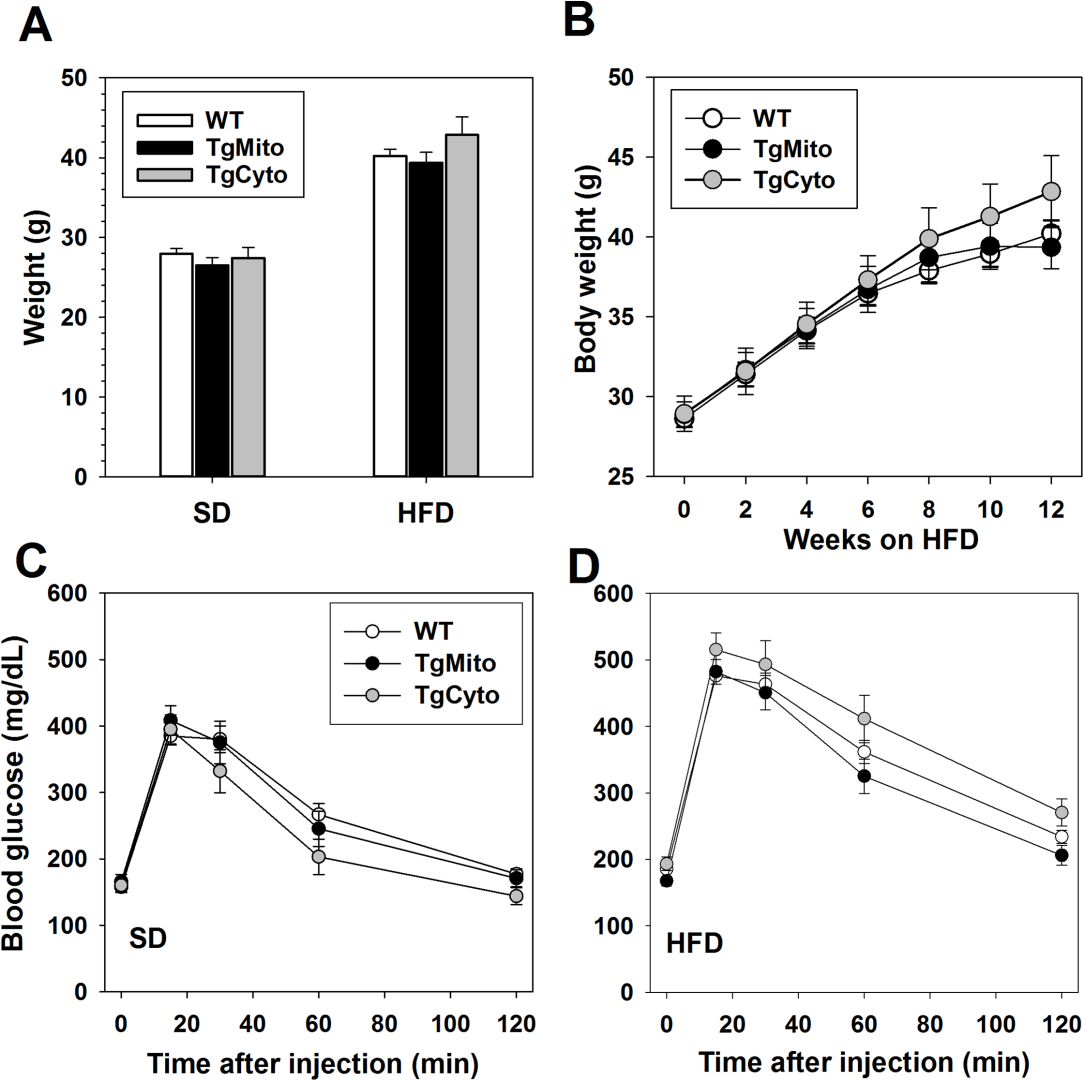
Overexpression of MsrA in the mitochondria prevents obesity-induced insulin resistance without altering weight gain. (A) Body weights of WT (n = 26), TgMito MsrA (n = 19), and TgCyto MsrA (n = 12) mice fed standard rodent chow (SD) or high fat diet (HFD). Bars represent mean body weight ± SEM. (B) Body weight gain of mice in (A) with HFD feeding. (C) Glucose tolerance tests of WT (n = 22), TgMito MsrA (n = 13), and TgCyto MsrA (n = 7) maintained on standard rodent chow (SD) or high fat diet (HFD). Circles represent mean body weight ± SEM.

In adult male mice maintained on standard rodent chow (SD), body weights of both transgenic lines did not differ from control (WT) mice (**Fig 1**). When fed HFD, both TgCyto and TgMito lines gained weight on a similar trajectory as WT mice and after 12 weeks of feeding we found no significant difference among the three lines (**Fig 1**). At sacrifice, HFD-fed WT and TgMito mice did not differ in fat distribution as measured by the weights of each individual fat depot, but TgCyto subcutaneous and brown fat depots were slightly, though significantly, larger than both (**Table 1**).

**Table 1:** Weight (±SE) of adipose tissue depots in HFD fed mice.

Adipose depot	WT (g)	TgMito MsrA (g)	TgCyto MsrA (g)
Epigonadal	2.23 (0.08)	2.16 (0.11)	2.31 (0.12)
Perirenal	1.16 (0.08)	1.16 (0.15)	1.31 (0.16)
Mesenteric	0.89 (0.11)	0.87 (0.15)	0.61 (0.18)
Inguinal subcutan.	**1.38 (0.09)** ^**a,b**^	**1.02 (0.13)** ^**a**^	**1.65 (0.12)** ^**b**^
Subscapular subcutan.	0.71 (0.05)	0.68 (0.05)	0.74 (0.07)
Brown	**0.27 (0.02)** ^**a**^	**0.24 (0.03)** ^**a**^	**0.37 (0.02)** ^**b**^

Numbers highlighted in bold represent p < 0.05 as determined by ANOVA. Values indicated by highlighted letters (a or b) indicate groups that do not differ statistically as analyzed post-hoc.

**Fig 2:**
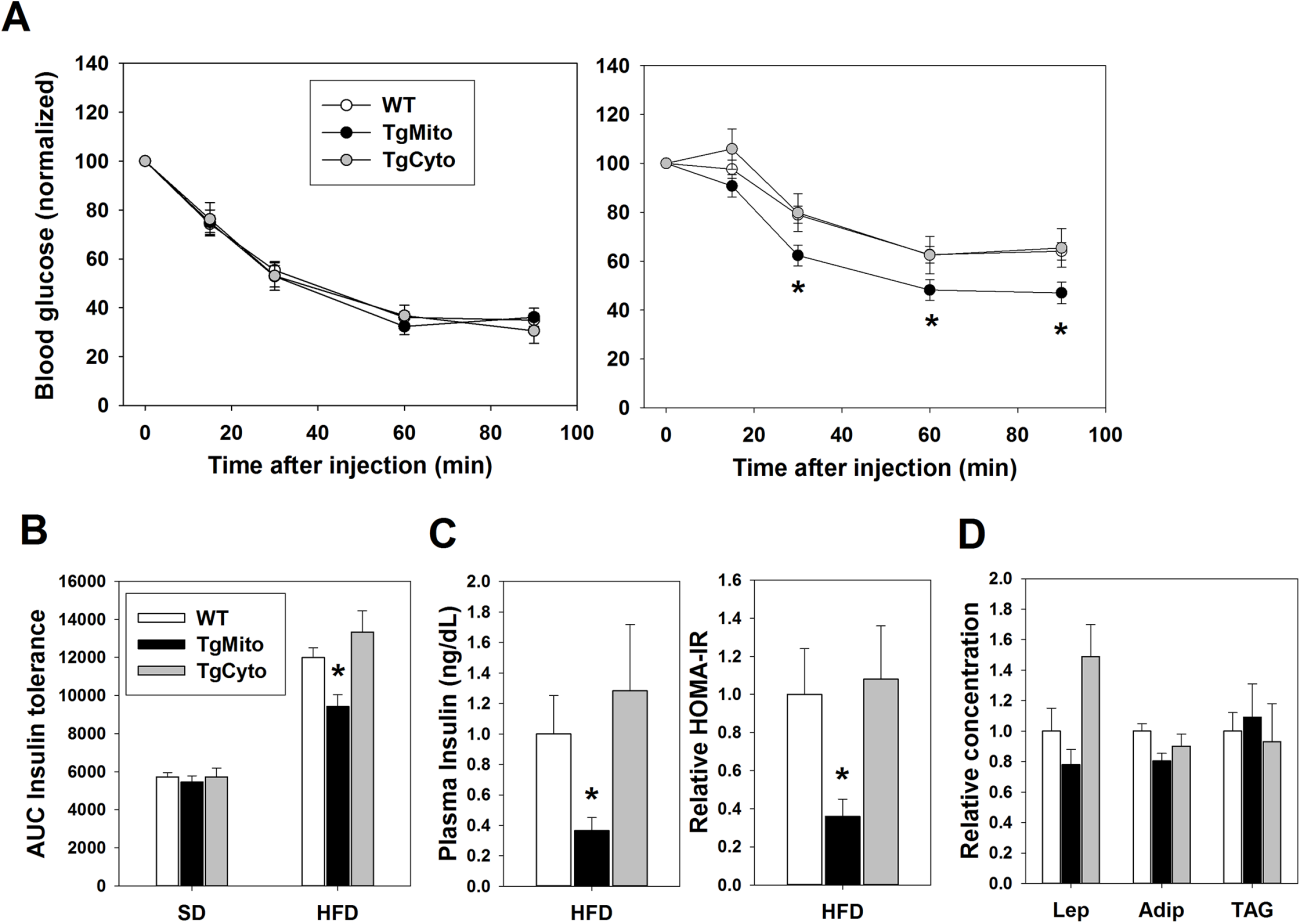
Mitochondrial MsrA overexpression prevents insulin resistance and hyperinsulinemia caused by obesity. (A) Insulin tolerance tests of WT (n = 22), TgMito MsrA (n = 13), and TgCyto MsrA (n = 7) mice maintained on standard rodent chow (SD) or high fat diet (HFD). All values for each genotype are normalized to the fasting blood glucose level of that genotype prior to injection of insulin. Asterisks represent p<0.05 by one-way ANOVA. (B) Area under curve (AUC) calculated for Insulin tolerance tests in Fig 1. Bars represent mean AUC x SEM. (C) Fasting (6 hour) plasma insulin concentration and HOMA-IR (normalized to mean WT value) of indicated mice maintained on high fat diet. (D) Relative plasma concentrations of leptin (Lep), adiponectin (Adip), and triglycerides (TAG) from overnight fasted, high fat diet-fed animals (values normalized to mean WT levels for each). Bars represent mean values ± SEM. For all, asterisks represent p<0.05 by one-way ANOVA.

HFD-feeding caused glucose intolerance among all three genotypes of mice; however, there was no effect of genotype on glucose tolerance in mice fed either SD or HFD (**Fig 1**). Similarly, there was no difference in insulin response as measured by insulin tolerance tests among the three lines when mice were maintained on a low-fat SD (**Fig 2**). Mice fed a HFD showed reduced insulin sensitivity compared to their SD-fed counterparts (**Fig 2**). TgCyto MsrA and WT mice did not differ in the degree of insulin resistance caused by HFD feeding. In contrast, TgMito MsrA mice were significantly more insulin sensitive than both WT and TgCyto MsrA mice after HFD feeding, though still relatively insulin resistant compared to mice maintained on a SD (**Fig 2)**. Fasting (6 hour) plasma glucose concentrations did not differ among the three genotypes of HFD-fed mice (WT fasting glucose (± SEM) = 174 ± 5 mg/dL, TgMito MsrA = 164 ± 5 mg/dL, TgCyto = 189 ± 11 mg/dL). However, fasting (6 hour) insulin concentrations were significantly reduced in HFD-fed TgMito MsrA mice compared to both HFD-fed WT and TgCyto MsrA mice suggesting reduced need for insulin to maintain relative normal glucose levels (**Fig 2**). HFD-fed TgMito MsrA mice also showed a reduction in HOMA-IR index, indicative of the relative insulin sensitivity in this group of mice compared to the other two genotypes (**Fig 2**). The metabolic benefit of TgMito MsrA mice among HFD-fed mice was not associated with significant alterations in concentrations of leptin, adiponectin or triglycerides in the plasma measured after an overnight fast (**Fig 2**).

**Fig 3:**
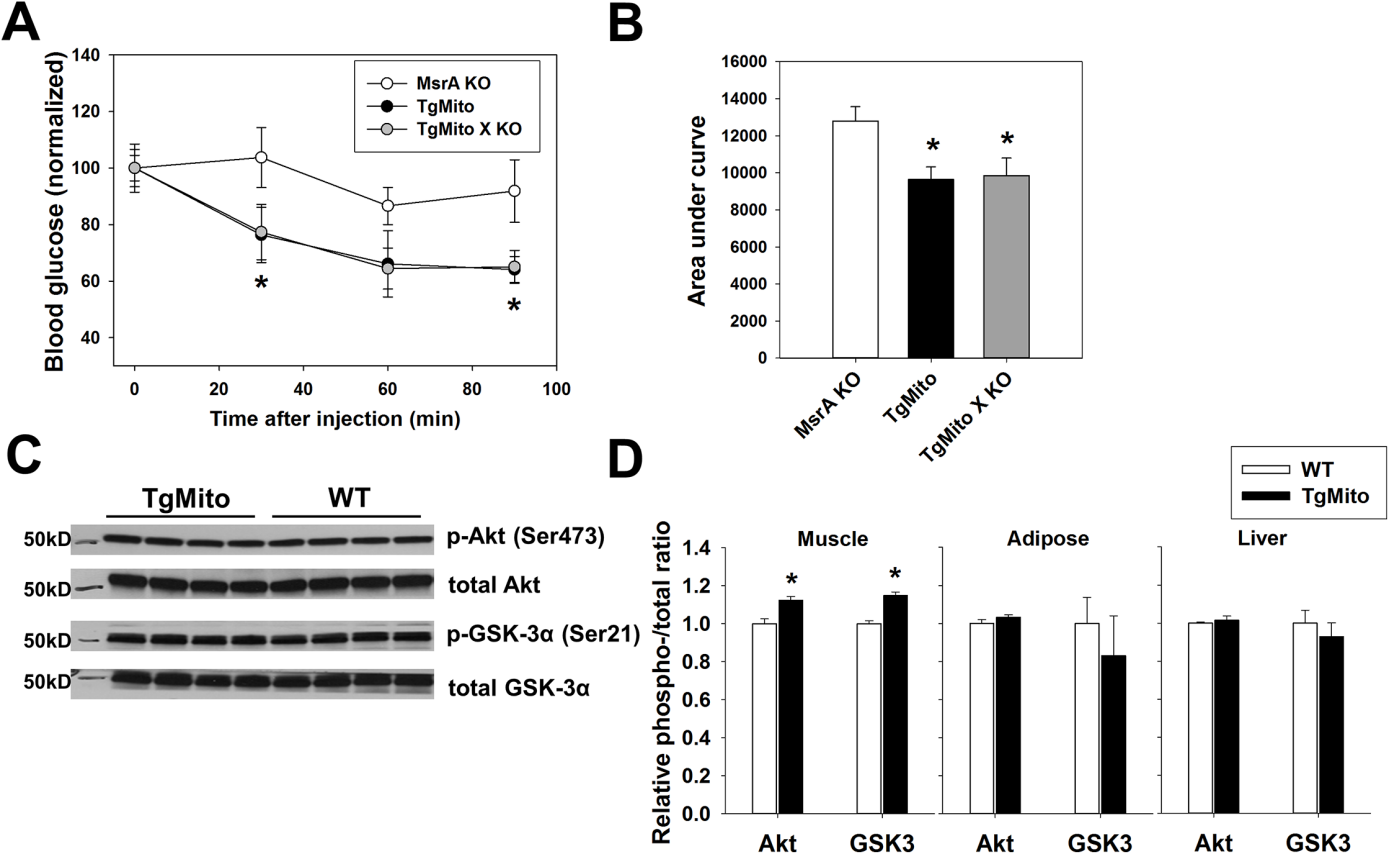
Increased MsrA in the mitochondria prevents insulin resistance even in the absence of cytosolic MsrA. (A) Insulin tolerance tests for HFD-fed MsrA KO (n = 6), TgMito MsrA (n = 4), and TgMito MsrA X KO (n = 7) mice. Circles represent mean blood glucose ± SEM. All values for each genotype are normalized to the fasting blood glucose level of that genotype prior to injection of insulin. (B) Area under curve (AUC) calculated for data in (A). Asterisks represent p<0.05 by one-way ANOVA (C) Representative blot of phosphorylation of Akt (Ser473) and GSK-3α (Ser21) in muscle (gastrocnemius) from HFD-fed mice. (D) Quantitation of phosphorylation of Akt and GSK-3α in muscle (gastrocnemius), visceral white adipose tissue (epigonadal) and liver. Values are normalized relative to mean WT value. Asterisks represent p<0.05 between genotypes by Student’s t-test. For all, bars represent mean AUC ± SEM.

Because TgMito MsrA mice still maintain endogenous MsrA expression in both the cytosol and mitochondria, we next tested whether mitochondrial MsrA overexpression even in the absence of cytosolic MsrA could prevent HFD-induced metabolic dysfunction. To address this, we crossed TgMito MsrA mice to mice lacking MsrA (MsrA KO) to generate a line of mice carrying the mitochondrial-targeted MsrA transgene in an MsrA-null background (TgMito X KO). After feeding a HFD, we found that both TgMito MsrA and TgMito X KO mice remained relatively more insulin sensitive compared to MsrA KO mice (**Fig 3**). Moreover, we found no evidence for any difference in insulin sensitivity between TgMito MsrA and TgMito X KO lines of mice. These results suggest that the metabolically beneficial phenotype of TgMito MsrA mice is due solely to the mitochondrial overexpression of MsrA and not to any potential interaction with the endogenous cytosolic MsrA.

Several tissues, including muscle, liver and adipose tissue, contribute to the regulation of glucose concentrations in response to insulin by activation of insulin signaling to promote glucose uptake. After HFD-feeding, insulin-stimulated activation (phosphorylation) of Akt and GSK-3α in was significantly reduced in muscle to SD-fed mice (**S2 Fig**). In line with our insulin tolerance test data, insulin-stimulated phosphorylation of Akt and GSK-3α was relatively high in gastrocnemius muscle from HFD-fed TgMito MsrA mice as compared to HFD-fed WT suggesting a relative preservation of insulin signaling in this tissue (**Fig 3**). Surprisingly, visceral adipose (epigonadal depot) and liver showed no difference in these markers at the resolution of this assay suggesting that increased mitochondrial MsrA may not prevent HFD-induced insulin resistance in these tissues (**Fig 3**). This might partly be due to the relatively higher mitochondrial content of muscle relative to liver and fat. Based on these findings, we focused the remainder of our study on skeletal muscle. Among SD-fed mice, there was no difference in insulin activation in this tissue (**S1 Fig**), suggesting that the overexpression of mitochondrial MsrA helps to prevent insulin resistance caused by metabolic stress, rather than generally improving insulin signaling under all conditions.

### Overexpression of mitochondrial MsrA does not alter mitochondrial content but activates AMPK signaling in obese mice

**Fig 4:**
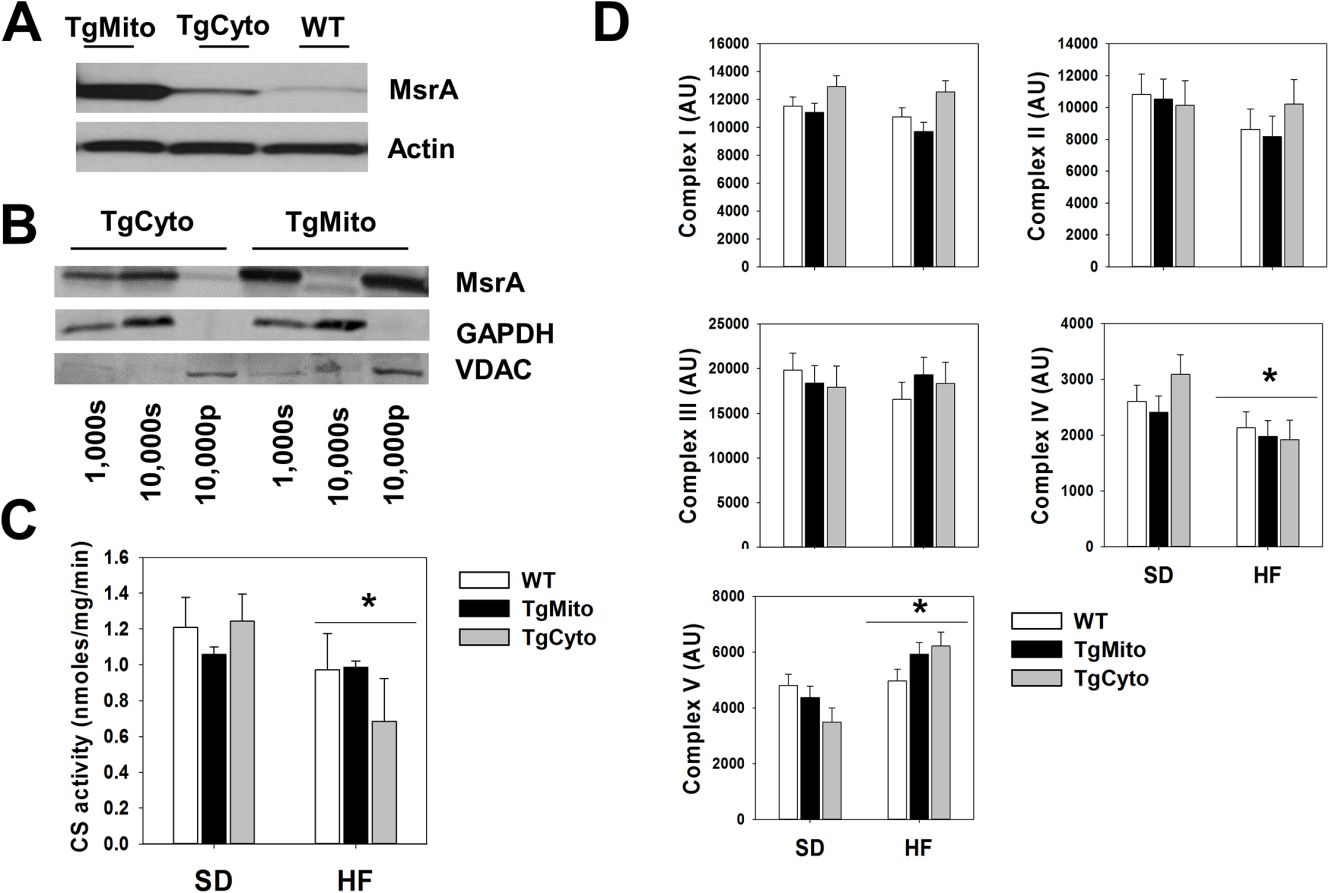
Overexpression of mitochondrial MsrA does not alter mitochondrial content. (A) Representative blot of MsrA expression in muscle. (B) Representative blot of subcellular distribution of MsrA in muscle. (1,000s) = 1,000 x g supernatant, 10,000s = 10,000 x g supernatant, 10,000p = 10,000 x g pellet. (C) Citrate synthase activity in muscle from SD- and HFD-fed mice. Asterisks and horizontal line indicate significant effect of diet as measured by two way ANOVA. (D) Quantification of western blots for Complex I subunit NDUFB8 (Complex I), Complex II subunit 30kDa (Complex II), Complex III subunit Core 2 (Complex III), Complex IV subunit I (Complex IV), and ATP synthase subunit alpha (Complex V). Values are given as arbitrary units (AU) normalized among groups to levels of VDAC. Bars represent mean (relative to values in WT) ± SEM. Asterisks and horizontal line indicate significant effect of diet as measured by two way ANOVA.

While endogenous MsrA is expressed in at relatively low levels in skeletal muscle [16], expression of MsrA is approximately 10–40 fold higher in muscle from both transgenic lines with overall expression of MsrA in the TgMito MsrA line greater than that found in mice of the TgCyto MsrA line (**Fig 4A**). Previous reports have suggested a similar level of overexpression in liver, heart, and fibroblasts derived from these lines of mice as well as relative increases in MsrA activity [19, 20]. We confirmed that the increased expression of MsrA in muscle of each line was targeted correctly to the intended cellular localization, *i*.*e*., increased MsrA was targeted to the mitochondria in TgMito MsrA mice and to the cytosol in TgCyto MsrA mice (**Fig 4**). Because TgMito MsrA mice were generated using the endogenous MsrA mitochondrial targeting sequence, it is likely that MsrA produced from the transgene is localized in its native location in the mitochondrial matrix [18–20, 22]. We first considered that increased MsrA may reduce levels of obesity-induced oxidative stress. As predicted, mice fed HFD showed an increase in oxidation of proteins (carbonyls) in skeletal muscle; however, carbonyls were not reduced in HFD-fed TgMito MsrA mice suggesting that this was not the likely mechanism for preserved insulin sensitivity in these mice (**S1 Supporting Information and S2 Fig**). We next considered that increasing MsrA in the mitochondria might promote mitochondrial biogenesis, but found that genotype had no effect on the activity of citrate synthase, a reliable estimate of relative mitochondria quantity, in the muscle of either SD- or HFD-fed (**Fig 4**). However, citrate synthase activity was significantly reduced (p = 0. 026) in muscle from HFD-fed mice relative to tissue collected from mice on SD. We also found that genotype did not alter relative expression levels of of subunits of electron transport chain complexes I-V on either SD or HFD (**Fig 4**). Prolonged feeding with HFD did significantly reduce expression of complex IV (p = 0.042) and increase expression of complex V (p = 0.002) relative to SD among all mouse strains.

**Fig 5:**
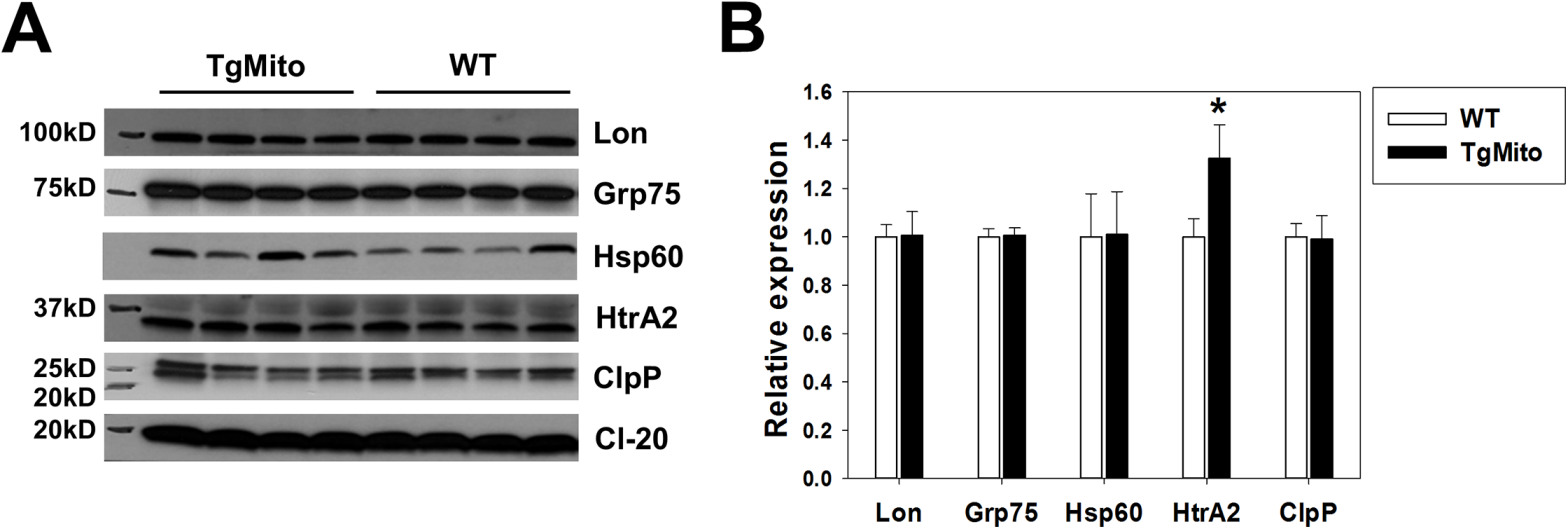
Mitochondrial-targeted MsrA overexpression does not alter expression of mitochondrial unfolded protein response in muscle from HFD-fed mice. (A) Representative blots for indicated proteins. Complex I subunit NDUFB8 (CI-20) was used as a loading control. (B). Quantification of (A). Bars represent mean (relative to mean values in WT) ± SEM. Asterisks represent p<0.05 between genotypes by Student’s t-test.

Because MsrA plays a primary role in protein oxidation repair, we addressed whether there was evidence that increasing mitochondrial MsrA might alleviate mitochondrial proteostatic stress. Obesity/HFD is associated with higher levels of oxidative damage, including damage to proteins [1–7]. Oxidized proteins can initiate the unfolded protein response (UPR), a cellular response to alleviate proteostatic stress. The mitochondria maintain an organelle-specific network (mitochondrial UPR) to regulate protein quality control [23]. We tested whether the improved insulin sensitivity of HFD-fed TgMito MsrA mice might be associated with modulation of this network by measuring levels of several of these components in muscle. These components included the ATP-dependent mitochondrial proteases Lon protease and ClpP; the serine protease HtrA2/Omi, and the mitochondrial protein chaperones Grp75, Hsp60. With the exception of modestly increased levels of HtrA2/Omi in TgMito MsrA muscle, there was little effect of mitochondrial MsrA on expression of mitochondrial UPR components in HFD-fed mice (**Fig 5**).

**Fig 6:**
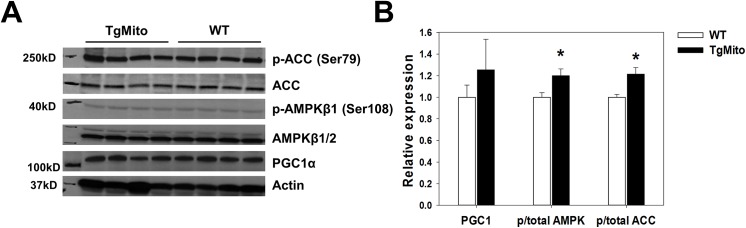
Increased mitochondrial MsrA promotes increased AMPK signaling in mice fed a high fat diet. (A) Representative blot assessing phosphorylation of ACC and AMPK and expression of PGC1α in muscle from HFD-fed mice. (B) Quantification of A. Bars represent mean (relative to mean values in WT) ± SEM. Asterisks represent p<0.05 between genotype by Student’s t-test.

We next tested whether overexpression of MsrA in the mitochondria might alter metabolic homeostasis that then improves insulin sensitivity under metabolic stress [24]. AMP-activated protein kinase (AMPK) is a cellular energy sensor that can up-regulate fatty acid oxidation in response to low levels of cellular ATP. We found evidence for increased activation of this pathway in muscle from HFD-fed MitoTg MsrA mice with increased phosphorylation of both the AMPKβ1 subunit, and a key downstream target of AMPK, Acetyl-CoA carboxylase (ACC), and upregulation (though not statistically significant) of the transcriptional regulator PPARγ coactivator-1 (PGC1) (**Fig 6**). Together, these data suggest that activation of this pathway in TgMito MsrA may at least in part mediate the improved insulin sensitivity of these mice under obese, HFD-fed conditions.

## Discussion

In this study, we show that increasing levels of the protein repair enzyme MsrA in the mitochondria, but not cytosol, improves metabolic dysfunction in mice rendered obese by feeding a high fat/high sugar diet. Both the cytosolic and mitochondrial forms of MsrA are translated from a single MsrA gene encoding 233 residues. Translation beginning from residue 1 of this gene encodes a mitochondrial targeting sequence whereas translation from residue 21 skips this targeting sequence and results in cytosolic MsrA [17]. Both the cytosolic and mitochondrial form of MsrA are equally capable of reducing methionine sulfoxide *in vitro* [17, 19]. Together with our previous study [16], we have now clearly identified a significant and novel role of MsrA, particularly in the mitochondria, in the regulation of glucose metabolism and insulin response in mice. Further examination of the benefits of mitochondrial MsrA against metabolic stress may be able to more accurately define these potential mechanisms.

While the finding that mitochondrial-targeted MsrA is intriguing, it is also counter to our initial prediction that cytosolic MsrA may be more beneficial. First, the normal distribution of MsrA in the mammalian cell is approximately three times greater in the cytosol than in the mitochondria [17, 18], suggesting the potential for a greater cytosolic need for this antioxidant repair enzyme. Second, we previously highlighted a role for MsrA in preserving cellular insulin response by preventing the accumulation of oxidative adducts on the insulin receptor, a trans-membrane protein that partially exists within the cytosol [16]. The lack of the repair of these adducts in MsrA KO mice contributes to their sensitivity to obesity-induced insulin resistance. However, our findings here show that replacing MsrA at high levels in the mitochondria alone abolishes the increased susceptibility to insulin resistance of MsrA KO mice. While we did not formally measure the oxidation of insulin receptor in this study, it seems unlikely that overexpression of MsrA in the mitochondria could directly modulate the oxidation repair of a protein found at the cellular membrane. Because the activity of MsrA is regulated primarily through the activity of thioredoxins, however, we cannot exclude the possibility that alterations in the redox environment of the mitochondria could not directly alter those in the cytosol. Thus, these data point to at least two different ways in which MsrA likely plays a role in the regulation of glucose homeostasis. Our results here do present a potential confound as the total expression level of MsrA is higher in the muscle of TgMito MsrA mice than it is in TgCyto MsrA mice. It is possible that our findings result from the higher levels of MsrA rather than its cellular location and this idea warrants further exploration.

Mitochondrial dysfunction and/or insufficiency has been speculated to be an underlying cause of the numerous metabolic dysfunctions that develop due to obesity [25, 26]. While MsrA is ubiquitously expressed both natively and in these transgenic mice, we found that protective benefit of mitochondrial MsrA overexpression may be primarily due to its effects in skeletal muscle. Because skeletal muscle has a fundamental role in the maintenance of normal glucose homeostasis, profound reductions in respiratory capacity, mitochondrial number, and impaired utilization of substrates of mitochondria in this tissue have been linked with obesity, insulin resistance, and T2DM [27, 28]. Intriguingly, the early transition to HFD has been associated with an increase in mitochondrial number and a shift towards preferential fatty acid oxidation capacity suggesting a potential compensatory response [29, 30]. However, chronic exposure to high levels of fatty acids, as would occur with the consumption of a HFD, disrupts mitochondrial fuel utilization and can result in incomplete β-oxidation of fatty acids and depletion of mitochondrial substrate intermediates [31]. We did find evidence that increased levels of mitochondrial MsrA were associated with elevated activation of the AMPK pathway. Activation of this pathway has been shown elsewhere to promote mitochondrial fatty acid oxidation [24, 32]. Further studies will be required to address the potential role mitochondrial MsrA in this pathway.

The normal function of MsrA in the mitochondria is still a matter of debate. While no direct role for MsrA has yet been noted in the regulation of mitochondrial energetic function, the lack of this enzyme in yeast and mammalian cells produces various different forms of mitochondrial dysfunction [33, 34]. In the mitochondria, MsrA can directly interact with the ETC complexes *in vivo* and prevents oxidation and aggregation of the ETC-associated protein cytochrome C following treatment with oxidative stress [35]. Similarly, the lack of MsrA in mice has been associated with increased oxidation of the cardiac mitochondrial proteome [36]. Intriguingly, the mitochondrial proteome is relatively rich in methionine residues compared to the cytosolic proteome, suggesting these proteins are at greater risk for methionine oxidative damage [37]. Moreover, the import of most mitochondrial proteins from the cytosol occurs in an unfolded state that is highly susceptible to oxidation [38]. It is interesting to note that MsrA preferentially repairs oxidized methionines in unfolded proteins, suggesting MsrA has a chaperone-like role in maintaining proteostasis [39]. While we did not find strong evidence that mitochondrial MsrA dramatically alters the mitochondrial UPR, a careful address of how obesity and mitochondrial MsrA affect the oxidation of the mitochondrial proteome could enlighten this field.

Part of our rationale for this study was based on reported associations between single nucleotide polymorphisms within a chromosome region containing the human MSRA loci and the incidence of obesity and metabolic syndrome in human populations [11–14]. In support, plasma levels of methionine sulfoxide are relatively increased in patients diagnosed with diabetes [40, 41]. However, a significant question that remains regarding the biological rationale for these relationships. One possibility could be that oxidation of methionine residues, and their subsequent reduction by MsrA, acts as a free-radical sink to reduce the potential for damaging other, potentially more critical, amino acid residues in cellular proteins as has been proposed by Levine *et al*. [42, 43]. The association between MsrA and insulin resistance might simply reflect alterations in the cells ability to utilize this “last-chance” antioxidant defense system for proteins. Our results suggest that reducing the oxidative stress and subsequent protein oxidative damage in the mitochondria, and not the cytosol, may play a critical role in preventing obesity-induced metabolic disease, as has been previously reported [4, 7].

On the other hand, the reversibility of methionine oxidation through methionine sulfoxide reductases suggests its potential as a cellular regulatory moiety akin to phosphorylation or acetylation. The oxidation of a single methionine residue to methionine sulfoxide in a voltage-gated potassium channel inactivates the function of this channel [44]. Moreover, expression of MsrA sufficiently reduces this methionine sulfoxide and re-activates the potassium channel. The activity of a key mitochondrial regulatory protein, calcium/calmodulin-dependent protein kinase II (CAMKII), is also regulated by methionine oxidation [36]. CAMKII regulates the mitochondrial calcium uniporter which controls the Ca^2+^ uptake and sequestration that are critical for regulating ATP production through the citric acid cycle [45]. Oxidation of CAMKII at key methionine residues activates this protein and interferes with the interaction of the autoinhibitory and catalytic domains; activation is ablated by replacing these methionine residues with valines [36]. CAMKII oxidation is significantly increased in the hearts of MsrA KO mice and leads to increased apoptosis and reduced survival following myocardial infarct surgery [36]. Diabetes is also associated with increased oxidation of CAMKII and transgenic mice generated with a methionine oxidation resistant CAMKII are able to maintain normal cardiac function even with diabetes [46]. What role CAMKII may play in the development of skeletal muscle insulin resistance remains to be addressed; however, CAMKII has been implicated in mediating the effects of ER stress, which itself has been linked as a driver of insulin resistance [47, 48]. Interestingly, MsrA has been shown to have the ability to both catalytically reduce as well as catalytically oxidize methionine through a reversal of the reduction process [49]. This raises the intriguing possibility that the activation/inactivation of proteins such as CAMKII may be directly regulated by MsrA rather than mediated through passive oxidation processes.

Identifying mechanisms by which some of those clinically classified as obese remain metabolically healthy could provide targets for therapeutic approaches to benefit those at-risk for metabolic diseases. Increasing mitochondrial MsrA recapitulates some of these phenotypes in that the mice are obese but remain insulin sensitive. While previous studies have largely shown limited effects of antioxidant therapy in diabetes, development of antioxidants targeted specifically to the mitochondria have shown promise in rodent models [4, 7]. Moreover, because MsrA is capable of repairing a particular form of oxidative damage, using this model might be capable of identifying specific proteins and/or residues to which these strategies could be targeted. Such therapeutic approaches could then bring about the promise of reversing the effects of T2DM or other metabolic disorders rather than simply addressing their symptoms.

## Supporting Information

S1 Fig Overexpression of MsrA does not alter insulin signaling in muscle from chow-fed mice. (A) Representative blot of phosphorylation of Akt (Ser473) and GSK-3α (Ser21) in muscle (gastrocnemius) from SD- and HFD-fed mice. Mito = TgMito, Cyto = TgCyto. (B) Quantitation of phosphorylation of Akt and GSK-3α in muscle (gastrocnemius) from SD-fed WT, TgMito and TgCyto mice. For all, bars represent mean ratio of phosphorylated:total protein levels ± SEM.
(TIF)

S2 Fig Overexpression of MsrA does not prevent oxidative damage in muscle of high fat fed mice. Carbonyl levels in muscle from mice of indicated genotype and diet. Bars represent average value for 3–5 animals; error indicated is standard deviation. Values are normalized to average value for WT chow diet. Chow = chow diet. HFD = high fat diet. Significant effect of diet, but no effect of genotype as measured by ANOVA.
(TIF)

S1 Supporting Information Methods provided for carbonyl assay presented in S2 Fig. (DOCX)
