# Supplementary figures and images for: MsrA Overexpression Targeted to the Mitochondria, but Not Cytosol, Preserves Insulin Sensitivity in Diet-Induced Obese Mice
Source: PLoS One. 2015 Oct 8;10(10):e0139844. doi: 10.1371/journal.pone.0139844 (PMC4598006; doi:10.1371/journal.pone.0139844)

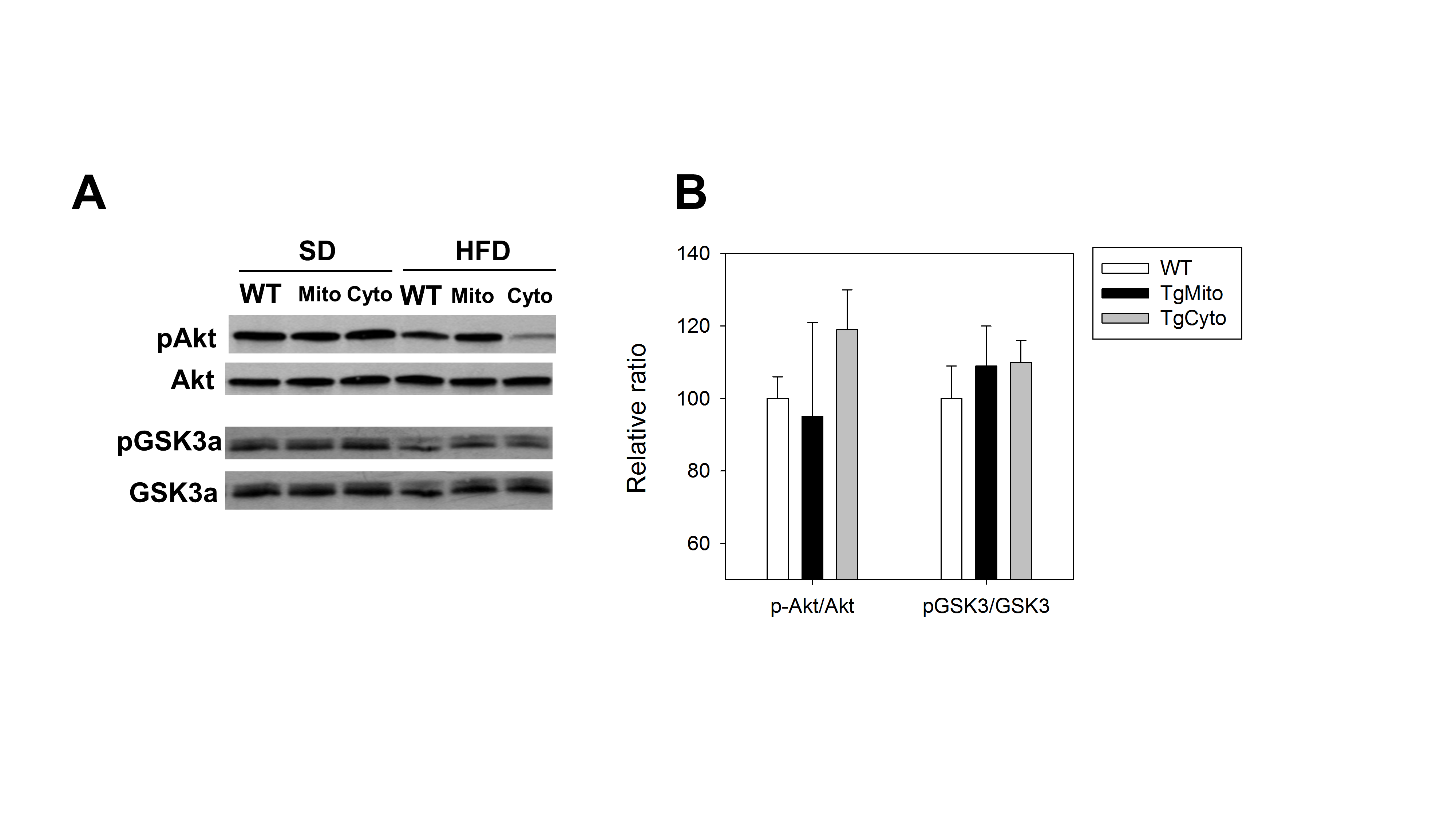

Supplement: S1 Fig — (A) Representative blot of phosphorylation of Akt (Ser473) and GSK-3α (Ser21) in muscle (gastrocnemius) from SD- and HFD-fed mice. Mito = TgMito, Cyto = TgCyto. (B) Quantitation of phosphorylation of Akt and GSK-3α in muscle (gastrocnemius) from SD-fed WT, TgMito and TgCyto mice. For all, bars represent mean ratio of phosphorylated:total protein levels ± SEM. (TIF) [file pone.0139844.s001.tif]
