# Supplementary material for: MsrA Overexpression Targeted to the Mitochondria, but Not Cytosol, Preserves Insulin Sensitivity in Diet-Induced Obese Mice
Source: PLoS One. 2015 Oct 8;10(10):e0139844. doi: 10.1371/journal.pone.0139844 (PMC4598006; doi:10.1371/journal.pone.0139844)
Supplement: S1 Supporting Information — (DOCX) [file pone.0139844.s003.docx]

**S1 Supporting Information**

**Carbonyl Assay.** Protein carbonyls in total skeletal muscle cellular extracts were determined using the method described in [16]. Snap-frozen tissues were homogeneized ina buffer containing 50mM phosphate pH 6.0, 0.5mM MgCl_2_,10 mM EDTA and a cocktail of protease inhibitors including phenylmethanesulfonyl fluoride (0.5mM), leupeptin (1mg/mL) and aprotinin (1mg/mL). After homogenization, extracts were centrifuged at 4°C, 100,000 g for 1 hour followed by incubation with 1% streptomycin sulfate at 37°C for 30 minutes and a further centrifugation at 16,000 g for 10 min. Extracts containing soluble proteins were incubated 2 hour at 37°C in the dark with 1mM fluorescein 5-thiosemicarbazide in de-aerated phosphate buffe, pH 6.0, with 0.3 M guanidine. Labelled proteins were resuspended with Trichloracetic acid, washed and resuspended in 20 mM phosphate buffer,pH 8.0,containing 8 M urea,and then boiled in Laemmli buffer. After separation by SDS-PAGE, fluorescence was visualized in gel using a Typhoon 9400 (excitation 532 nm,emission 526 nm,40-nm band pass; GE Healthcare LifeSciences, Piscataway, NJ, USA). Gels were then stained with coomassie blue, destained and imaged to normalize for protein amount.
